# Harder, better, faster, stronger: Colistin resistance mechanisms in *Escherichia coli*

**DOI:** 10.1371/journal.pgen.1009262

**Published:** 2021-01-07

**Authors:** Axel B. Janssen, Willem van Schaik

**Affiliations:** 1 Department of Medical Microbiology, University Medical Centre Utrecht, Utrecht University, Utrecht, the Netherlands; 2 Institute of Microbiology and Infection, University of Birmingham, Birmingham, United Kingdom; Institut Pasteur, CNRS UMR 3525, FRANCE

The antibiotic colistin (polymyxin E) is an amphipathic, non-ribosomally synthesized, cyclic lipopeptide, which is selectively bactericidal for gram-negative aerobic bacilli, as it targets lipopolysaccharide (LPS) molecules in their outer membranes [1]. Colistin first acts by replacing Ca^2+^ and Mg^2+^ cations that stabilize the outer membrane through electrostatic interactions with the anionic phosphate groups of the lipid A moiety of LPS [2]. Colistin then inserts itself into the outer membrane, negatively affecting the integrity of this barrier. However, destabilization of the outer membrane by colistin may not be lethal to the bacterial cell [3]. Indeed, the bactericidal activity of colistin appears to be primarily mediated by the permeabilization of the inner membrane through interactions between colistin and the LPS molecules that are located in the outer leaflet of the inner membrane after synthesis in the cytoplasm [4].

**Fig 1:**
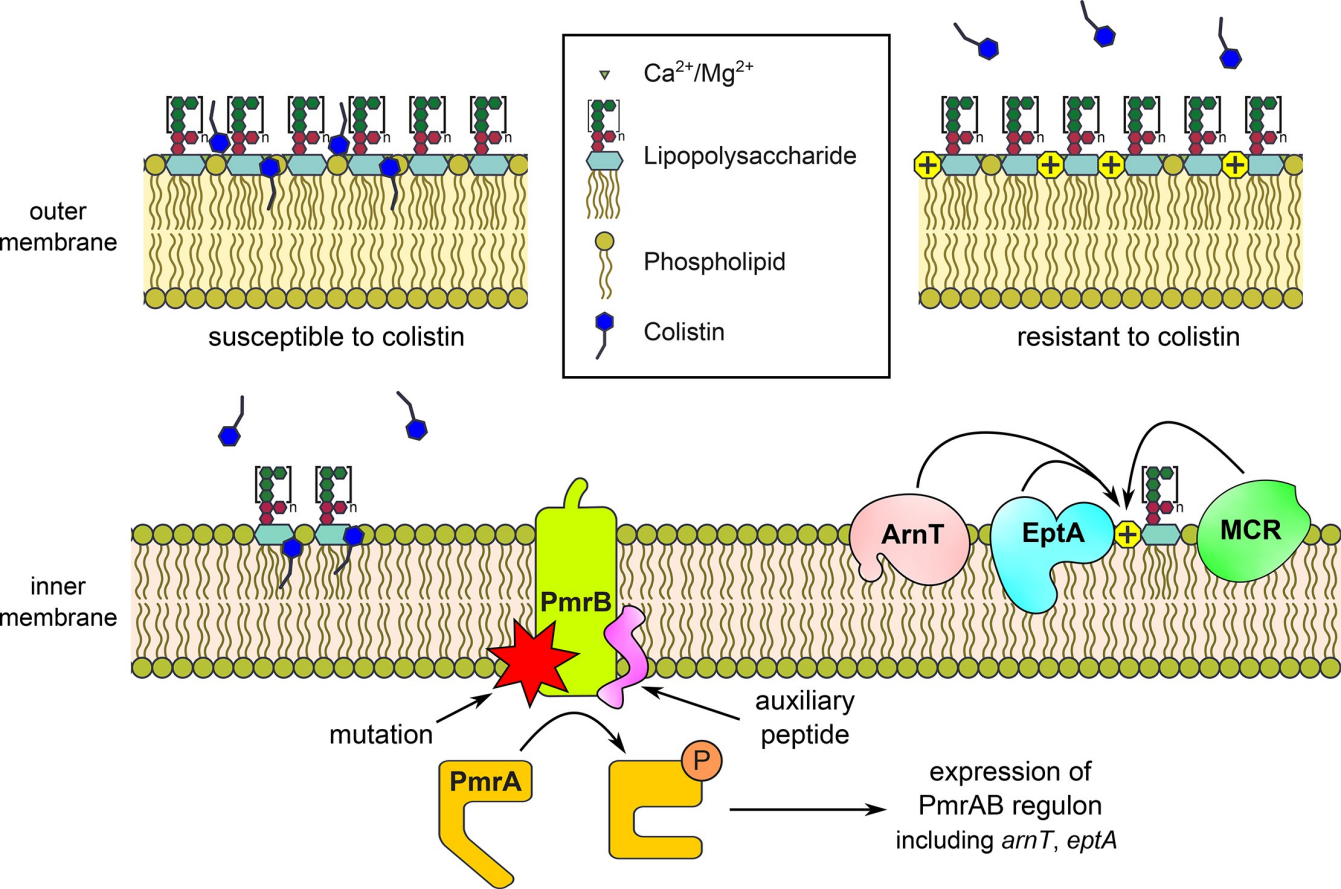
Colistin susceptibility and resistance in *E*. *coli*. Colistin initially targets LPS in the outer membrane of *E*. *coli* through electrostatic interactions with negatively charged phosphate groups on the lipid A moiety of LPS. After destabilizing the outer membrane, colistin binds to LPS molecules that are located in the outer leaflet of the cytoplasmic membrane while they await transport to the outer membrane. The resulting disruption of the inner membrane is proposed as the main cause of cell death. Previous studies in *E*. *coli* have revealed that mutations in the sensor histidine kinase PmrB are an important mechanism of colistin resistance, leading to the constitutive production of the enzymes ArnT and EptA that add a positive charge (4-amino-4-deoxy-L-arabinose and phosphoethanolamine, respectively) to the phosphate groups of lipid A, thereby reducing the affinity of colistin to lipid A. An alternative pathway toward colistin resistance is provided by acquisition of mobile genetic elements that carry *mcr* genes which also leads to the decoration of lipid A with phosphoethanolamine. Knopp and colleagues [12] show that novel auxiliary peptides can also cause colistin resistance through direct interactions with PmrB, leading to the activation of the two-component system. LPS, lipopolysaccharide; *mcr*, mobilized colistin resistance.

Because of the rapid increase in infections caused by multidrug-resistant strains of the family Enterobacteriaceae, colistin is now increasingly used as an antibiotic of last resort, and its use is increasing globally [5]. The increasing importance of colistin has drawn attention to mechanisms of acquired colistin resistance in important multidrug-resistant opportunistic pathogens, like *Escherichia coli* and *Klebsiella pneumoniae* [6]. While a variety of resistance mechanisms have been described, the most commonly encountered are those that lead to modifications of the lipid A moiety that reduce the affinity of colistin to lipid A or inhibit its successful insertion into the outer membrane (Fig 1). These changes can be mediated by the acquisition of mobilized colistin resistance (*mcr*) genes, of which 10 homologues have so far been described [7,8], or the accumulation of mutations that lead to an increased expression of chromosomal genes that mediate lipid A modifications [9]. Mutations in the two-component regulatory system PmrAB (also termed BasRS in *E*. *coli*) appear to be one of the most prominent causes for colistin resistance in clinical isolates of *E*. *coli* [10,11].

In this issue of *PLOS Genetics*, Knopp and colleagues describe an entirely novel mechanism by which *E*. *coli* can acquire resistance to colistin [12]. The authors of this study generated a library of *E*. *coli* clones that expressed more than 500 million random peptides of 10 to 50 amino acids in length. The clone libraries were then screened to identify peptides that conferred resistance to colistin. A total of 6 peptides, without any sequence homology to each other or to other proteins in public databases, were found to confer resistance to colistin to *E*. *coli*. Importantly, all peptides act as activators of the PmrAB (BasRS) two-component system, leading to the up-regulated production of enzymes that catalyze modifications of lipid A by 4-amino-4-deoxy-L-arabinose (by ArnT) and phosphoethanolamine (by EptA) (Fig 1). The authors provide evidence that their peptides directly interact with the sensor protein kinase PmrB, thus leading to constitutive activation of the regulatory system and increased expression of EptA, ArnT, and other genes that are controlled by the response regulator PmrA. Auxiliary peptides that directly interact with sensor histidine kinases appear to be common and most appear to suppress the activity of its cognate histidine kinase [13], but peptide activators, like the small (65 amino acids) transmembrane protein SafA, which interacts directly with the sensor histidine kinase PhoQ to activate the PhoPQ system, have also been described in *E*. *coli* [14]. The findings from Knopp and colleagues thus further expand our view on the potential for the evolution of novel auxiliary regulators of bacterial two-component regulatory systems.

It may be a matter of debate to what extent the findings of Knopp and colleagues [12] are relevant to understand the emergence of colistin resistance in clinical settings. Only 6 peptides were found among a library containing hundreds of millions of peptides. In addition, these peptides were expressed under the control of a very strong promoter, which is unlikely to be widespread in nature. In this respect, this work is in line with previous studies that showed that novel functions, including antibiotic resistance, can emerge from random sequence space, but do so at a very low frequency [15,16].

It is, however, important to note that the increased use of colistin in clinical and veterinary settings may be a powerful driving force that will strongly select for novel resistance determinants in multidrug-resistant gram-negative bacteria [17]. Indeed, this has been compellingly demonstrated by the emergence of the *mcr-1* gene in gram-negative bacteria [18]. This gene was first described in *E*. *coli* isolated in China in 2015 and encodes a membrane-associated enzyme that catalyzes the modification of phosphate residues on lipid A by phosphoethanolamine, thereby reducing the negative charge and reducing electrostatic interactions between lipid A and colistin [18]. Remarkably, *mcr-1* appears to have been mobilized only once in the mid-2000s and then rapidly expanded globally, with colistin use in agricultural settings probably being the main driver for its spread [19]. Similar selective pressures may drive the wide dissemination of peptides that activate PmrB. Notably, the fitness costs of the colistin resistance peptides identified by Knopp and colleagues were low [12], which could suggest that functionally similar peptides have the potential to spread as efficiently among gram-negative bacteria as *mcr-1*. Indeed, it is tempting to speculate that colistin-resistant clinical *E*. *coli* isolates in which no resistance mechanism could be identified [10,11] may already be carrying functionally similar peptides. There is thus a need for further functional studies into *E*. *coli* and other gram-negative bacteria with acquired colistin resistance with the aim to uncover novel mechanisms by which resistance to this last-resort antibiotic can emerge. The relative ease by which colistin resistance can be acquired by *E*. *coli*, either through mutation or horizontal gene transfer, is a cause for concern and should inform strict antimicrobial stewardship policies in human and veterinary medicine, to safeguard this increasingly important antibiotic for future use.
